# COVID-19 in a Pre-Omicron Era: A Cross-Sectional Immuno-Epidemical and Genomic Evaluation

**DOI:** 10.3390/vaccines11020272

**Published:** 2023-01-27

**Authors:** Jorge Pamplona Pagnossa, Sarah de Oliveira Rodrigues, Gabriel Ferrari de Oliveira, Mohd Adnan, Maryam Saud Aljaid, Isabela Bacelar de Assis, Alex Sandro Gomes Lima, Mitesh Patel, Hanan A. Ogaly, Gaber El-Saber Batiha

**Affiliations:** 1 Department of Biological Sciences, Pontifical Catholic University, PUC-Minas, Poços de Caldas 37714-620, Brazil; 2 Department of Electrical Engineering, Pontifical Catholic University, PUC-Minas, Poços de Caldas 37714-620, Brazil; 3 Department of Biology, College of Science, University of Hail, P.O. Box 2440, Saudi Arabia; 4 Department of Pediatrics, College of Medicine, Taif University, Taif 21944, Saudi Arabia; 5 Municipality of Cruzeiro, São Paulo 12701-450, Brazil; 6 Department of Biotechnology, Parul Institute of Applied Sciences and Centre of Research for Development, Parul University, Vadodara 391760, India; 7 Chemistry Department, College of Science, King Khalid University, Abha 61421, Saudi Arabia; 8 Biochemistry and Molecular Biology Department, Faculty of Veterinary Medicine, Cairo University, Giza 12211, Egypt; 9 Department of Pharmacology and Therapeutics, Faculty of Veterinary Medicine, Damanhour University, Damanhour 22511, Egypt

**Keywords:** COVID-19, serological tests, symptomatology, genome, SARS-CoV-2

## Abstract

The seventh human coronavirus was discovered and reported primarily in Wuhan, China. After intense seasons with repercussions in all areas of humanity, the pandemic demonstrates a new perspective. In Brazil, the pandemic concept had impacts in vast areas, including healthcare hospitals. This present study aims to describe and synthesize data from a determined period from the year 2021 that correlate the symptoms of passive and/or active patients for COVID-19 and their respective results of IgG/IgM serological tests in hospitals in the city of Cruzeiro, São Paulo, Brazil. The form had been applied to 333 people and obtained conclusive results and several symptoms were presented; in addition, asymptomatic cases were also analyzed and directed in the genomic study of variants of concern, as well as vaccination data in the study region.

## 1. Introduction

In 2019, an outbreak of flu-like syndrome of unknown origin affected citizens in Wuhan City, Hubei Province, China. Subsequently, the viral infection spread uncontrolled throughout the world, being declared a pandemic by the WHO Director-General in 2020 [1]. Like other respiratory viruses, the transmission of SARS-CoV-2 occurred with high infectivity, mainly through the respiratory tract. Droplet transmission was the main known route, although aerosols could also be recognized as a form of transmission [2]. Due to its easy transmissibility, the virus has taken place in several countries around the world, including Brazil.

In the Brazilian context, the pandemic proved to be a challenge in social, political, and primarily human health actions. The impacts of the pandemic in Brazil are evidenced in the chaotic situation of the health system, which did not have priority throughout the pandemic period [3], causing scarce basic resources and full neglect. After one year of COVID-19 and disastrous management of the disease, Brazil had more than 11 million cases, 270,000 deaths, and the highest number of daily deaths due to COVID-19 in the world [4], which could have been avoided if the government system as a whole had better performance and administration.

Moreover, the notification rate in Brazil was 9.9%, in which the population did not have hospital resources of very high demand and did not acquire the minimum knowledge necessary to deal with the disease [5]. The inefficiency of communication to the population about the reliability of tests was commonly present, not emphasizing the functioning and importance of serological screening tests to the population.

However, certain procedures were still implemented, and some hospitals performed screening tests, even with few resources, the resilience of health professionals permeated the pandemic perspectives, and certain types of study were conducted within the context highlighted, such as the use of IgG and IgM tests [6].

Several types of tests were used to detect the immunological response against COVID-19 in humans, including IgM/IgG rapid tests performed at the hospital admission. The human body produces two main types of antibodies, immunoglobulin M (IgM) and immunoglobulin G (IgG), in response to an infection. IgM antibodies are produced shortly after viral antigen recognition, while IgG antibodies are subsequently produced to maintain the immune system with the immune memory needed for an effective response to a new infection [7].

In a contextual parameter, the global crisis caused by the virus permeates society more devastatingly in developing countries due to the precariousness of hospitals and health centers. As of April 2021, there were more than 140 million confirmed cases and more than three million deaths from COVID-19 worldwide [8].

Additionally, in the Brazilian context, the pandemic became a great challenge for hospital institutions in general; vaccination was still incipient, and knowledge about the resolution of symptoms was still being implemented. However, certain symptomatic patterns were characterized by the WHO [9], evidencing diagnoses and providing a greater margin of positive prediction. Given this, the collection of data in the hospital scope that had been performed between June and November 2021 demonstrates the frequency of symptoms in active or passive patients for viral immune response, who sought care in health institutions in specific regions of the city of Cruzeiro-São Paulo, Brazil.

Due to the panorama of controversies about denialism and the affinity for medications that promoted a false safety against the virus and prevention of symptomatology, so that commercial sectors would be benefited even if causing major sanitary chaos, only a cross-sectional analysis of the critical moment that would precede the highest peak of new infections recorded to date (the last two months of 2021 and early 2022) could report which gaps could be avoided for the emergence of new infections variants, such as omicron, in the symptomatologic, immunological, genomic, and social behavior and public health management aspects.

In this context, it was investigated at the time of admission of patients to the hospital unit, a pre-selection of volunteers who agreed to answer a questionnaire about symptomatic signals in which it was applied inclusively, seeking reliable results on the serological tests of rapid diagnosis, vaccination, as well as the correlation with the main symptoms presented by patients within the pandemic context.

Furthermore, the common genetic components of the SARS-CoV-2 variants were also evaluated and characterized to directly estimate the appropriateness of hygienic–sanitary and/or medicinal measures of an experimental nature in the hospital and community environment, since the lack of engagement of the population to the effective measures against the spread of the virus resulted in the emergence of new variants, notably more infectious and lethal.

Studies with the complexity of data and information, such as the present one, have not been carried out before, evidencing its importance in understanding pandemic times and all their genetic approaches and variants of the SARS virus. As the main objective, the data were collected and properly processed and examined, demonstrating correlations between the variants, the greater transmissibility promoted by the spike protein, the diagnostic variety by the highlighted symptoms, evaluation by entropy, genetics, amino acids, and nucleotides, resulting in a complete evaluation of the collected data.

## 2. Methodology

The present study occurred in the pandemic period described between June and November 2021 with the application of a simple model questionnaire, covering age, gender, and vaccination as general classifiers and 20 symptoms that could be optionally indicated between “yes”, “no”, or “I don’t know”. A total of 333 patients participated in the research. The questionnaires were applied at the hospital institution: Integrated Healthcare Center, located in the city of Cruzeiro, state of São Paulo, Brazil. In addition to this questionnaire, serological tests (qualitative immuno-lateral flow technology, Biomanguinhos laboratory, Fiocruz—Oswaldo Cruz Foundation, Rio de Janeiro, Brazil) were performed for the presence of IgG and IgM antibodies as a way to correlate the symptoms and immunological response of each individual. The symptoms mentioned in the questionnaire were the same as evidenced by the World Health Organization in the specific period described. The reported symptoms as possible answers were fever, sore throat, rhinorrhea, cough, shortness of breath, chills, vomiting, nausea, diarrhea, headache, itching, conjunctivitis, myalgia, joint pain, loss of appetite, loss of smell, loss of taste, nasal bleeding, fatigue, and seizures.

These data were collected, organized, and synthesized in specific graphs and tables as directly correlated with the IgG and IgM results of the performed serological tests. In addition, the present study conducted careful bibliographic research about the symptoms analysis, social impacts, historical context, and general theoretical basis in the pandemic context, using research sources, such as PubMed, Scielo, and Google Scholar.

For genomic evaluation, bioinformatics tools were used. In this stage of the research, computational resources, such as “Python” algorithms (developed by the authors) and the “Nextclade” application were used. The first Python program was responsible for collecting from the National Center for Biotechnology Information (NCBI) database the genome of all SARS-CoV-2 samples found in Brazil between June and November 2021 and organizing them into FASTA format. Until the date of the last execution of the script (late of October 2022), a total of 4900 strains were available in the database. Thus, the genomes were inserted into the “Nextclade” software that compared them to the genome of the original SARS-CoV-2 Wuhan strain and then provided a TSV file containing information about the mutations that occurred, besides the entropy of genome information and the phylogenetic tree. The second “Python” script was responsible for processing the TSV file generated by “Nextclade”.

The keywords highlighted in this analytical and descriptive epidemiological research were: “COVID-19”, “pandemic”, “analysis”, “population”, “hospitals”, and “SARS-CoV-2” and were used without exclusion criteria. Thus, articles that were in the pre-established period had greater attention and relevance for the greater applicability in the study.

## 3. Results and Discussion

### 3.1. Frequent Symptoms of the Analyzed Volunteers

The city of Cruzeiro is located in the state of São Paulo, has a total area of 305.7 km^2^, and its registered population in 2010 was 77,039 people, with an estimation of 82,572 until 2019 [10]. The panorama of the situation of the city’s health system shows indicators favorably above average compared to other municipalities in the country and the state, such as 14.05 deaths per 1000 live births and 0.1 hospitalizations per 1000 inhabitants due to diarrhea. Currently, the city has 31 health facilities covered by the Unified Health System (SUS), including the central hospital and an Integrated Health Center (CISA), where the function of a campaign hospital for the coronavirus pandemic in cases of low and medium complexity were performed [10].

**Figure 1:**
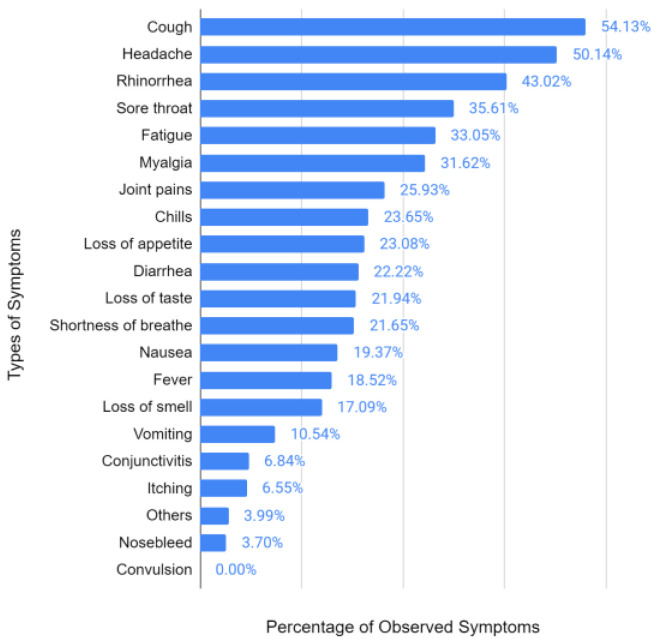
Frequency of symptoms in participants.

From the survey of the symptoms of people, with or without positivity for immunological response due to COVID-19 (Figure 1), respiratory tract involvement is the most prevalent symptom (cough: 54.13%; and rhinorrhea: 43.02%), followed by headache (50.14%), skeletal muscle disorders (fatigue: 33.05%; myalgia: 31.62%; and joint pains: 25.93%), and gastrointestinal (loss of appetite: 23.08%; and diarrhea: 22.22%). During the occurrence of a pandemic associated with a flu-like viral syndrome, the diversity of symptoms is a key factor in the patient’s decision to seek care at health centers to perform serological screening tests, or even to confirm the presence of the virus in the oropharyngeal tract.

The frequency of symptoms was correlated with the predictive symptoms most evident in the current literature. Recent studies have demonstrated the involvement of the naso-oropharyngeal tract as a possible typical symptom classifier for diagnosing cases of COVID-19, primarily evidencing the occurrence of cough (43.8%) [11].

**Figure 2:**
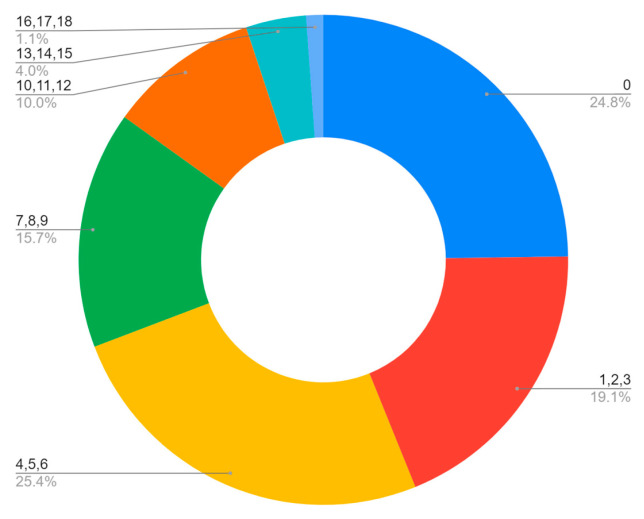
Number of symptoms per person.

Thus, it is emphasized that the symptomatology obtained different classifications, as well as the number of symptoms presented by each patient. After data processing, it was possible to observe (Figure 2) that 19.1% of the people presented only one, two, or three different symptoms and in greater quantity; 25.4% of the patients had four, five, or six symptoms, showing that the symptoms affected by the virus are mostly multiple. In addition, the number of symptoms between seven and twelve represents 25.7%, emphasizing the concept of symptomatic summation, which is based on primordial characteristic symptoms, which culminate in other symptoms of similar origins, physiologically and anatomically [12].

**Figure 3:**
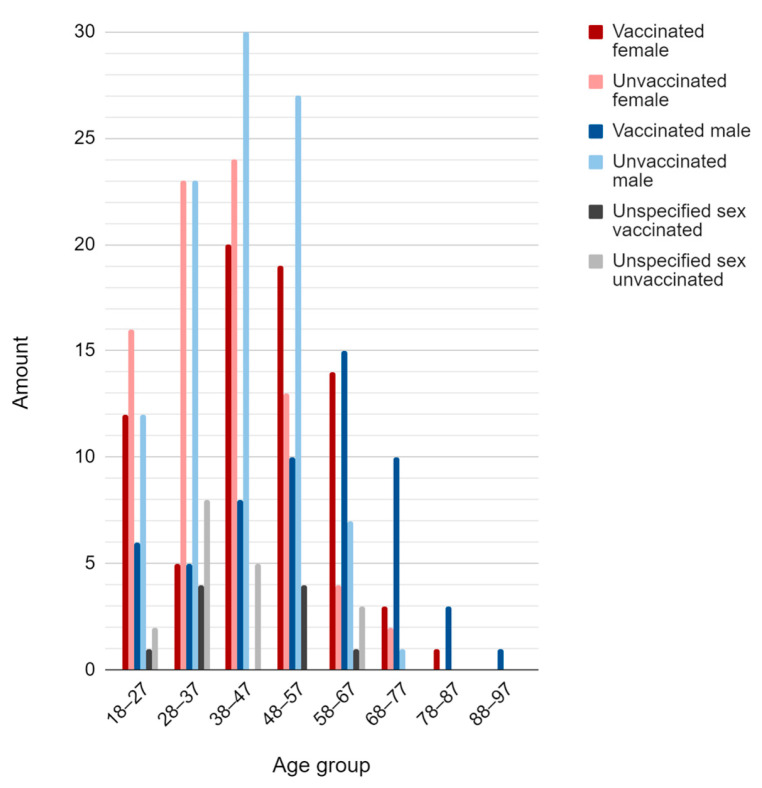
Amount of people divided into age group, vaccination, and sex.

In addition, the number of symptoms per person has direct proportionality with the number of patients present in the study and an essential numerical characteristic for the analysis of the group as a whole is the age factor (Figure 3).

During the specific period that the study was conducted, informative perspectives were implemented in the city of Cruzeiro-SP, decreed by the state of São Paulo, with a prescription of guidelines [13]. The instructions were to guide the population on hospital safety standards, being certain specific groups of higher priority in basic health units, seeking to avoid stocking and a greater probability of contamination and spread of the virus [14], both for patients and non-patients (positive and negative diagnoses). However, the visible highlight in age, obtained through data collected between July and November 2021, has its highest mean age between 35 and 60 years, evidencing 45 years specifically (Figure 3), as the age group most participating in the study. Demonstrating the correlation between age and prioritization of care for cases of specific or severe symptoms [15,16].

**Figure 4:**
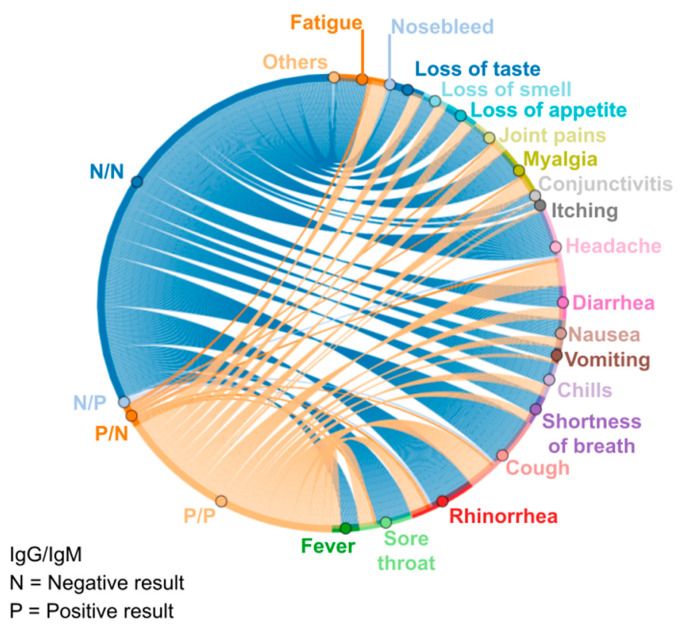
Symptoms correlated with positive and negative diagnoses of serological tests.

The public up to 60 years of age are among the highest percentage of the participants of the study because it was one of the current recommendations to seek care and diagnosis from symptomatic involvement; later, the patient was taken for medical evaluation, and after the procedure, serological tests were performed. In the large majority, only cases with probable severity were prioritized, excluding adolescents and children in the risk group. Data from serological tests correlated with the proportionality of symptoms reported by patients (Figure 4) were also processed, showing a higher frequency of negative diagnoses per symptom; that is, although participants reported symptoms, such as fatigue, diarrhea, and fever, the negative results in immunological tests indicate the possibility of involvement by flu-like syndromes similar to COVID-19. Symptoms are characteristic of other diseases, such as inflammation of the gastrointestinal tract of bacterial origin, for example.

It is possible to observe from Figure 4 that the share of positive results for the detection of IgG and/or IgM (N/N, negative for both tests; N/P, negative for IgG and positive for IgM; P/N, positive for IgG and negative for IgM; and P/P, positive for both tests) antibodies is quite representative of the symptomatic picture of the research participants. This fact shows not only the increase in cases of flu-like syndromes in this period of the year but also the unpreparedness of government agents and the population in general regarding the emergence of a health crisis of global proportions and high infectious and lethal potential [17]. Thus, it can be hypothesized that those problems in the public health system, such as overcrowding in hospitals, were aggravated by the lack of preventive measures in the dissemination of the SARS-CoV-2 virus, which culminated in the precariousness of care services for the neediest [18].

Another factor to be considered as adverse to the efforts against the dissemination and lethality of new strains of SARS-CoV-2 was the delay in the purchase and distribution of vaccines [19], a measure that would more efficiently mitigate the devastating effects on the Brazilian population. It is of great importance to emphasize that the diagnosis of COVID-19 does not imply the exclusion of other concomitant diseases. A list of viral and bacterial agents that cause symptoms similar to those of COVID-19 is shown in Table 1.

### 3.2. Genome Evaluation

As the pandemic exhibited high and rapid advance, there is a need to identify genetic risk factors, which can be fully harmful to humans due to their high capacity for mutation, hindering the possibilities of treatment and the creation of vaccines, evidencing the susceptibility of this serious disease [21]. Host genetic factors, along with other risk factors, can help determine susceptibility to respiratory tract infections [22]. Thus, the genomic analysis of the mutations that occurred in SARS-CoV-2 samples collected in Brazil between June and November 2021, about the original Wuhan strain was obtained through computational resources, as explained in the Methodology section [23].

**Figure 5:**
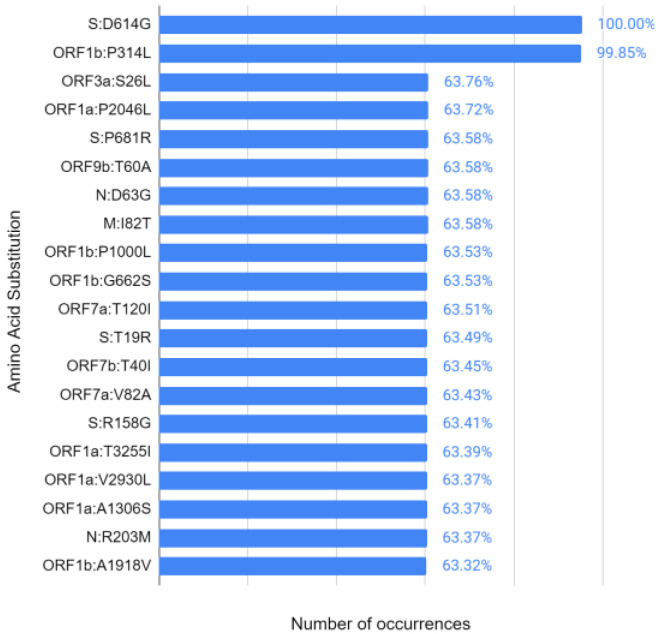
Most frequent amino acid substitutions in decrescent order.

The first analysis refers to the most frequent mutations in the strains analyzed. Thus, the 20 most frequent amino acid substitutions are presented in Figure 5 in descending order. The number of samples that had a given mutation is under the horizontal axis and the vertical axis indicates the gene and its mutation.

According to the results of similar studies [24,25], amino acid substitutions “S:D614G” and “ORF1b:P314L” are the most frequent, affecting approximately 100% of the analyzed samples. This indicates that the mutations found in Brazilian strains of SARS-CoV-2 accompany mutations found in strains from other parts of the world.

Regarding the mutation “S:D614G”, it occurs in position 614 of the spicule protein (S), where aspartic acid (D) is replaced by glycine (G). Protein S is responsible for linking the virus to the host cell through the angiotensin-converter enzyme 2 (ECA 2) [24]. Due to the function of protein S, studies indicate that the mutation “S:D614G” increases the transmissibility of the virus, as it makes the interaction between the virus and ECA 2 more efficient [25]. Although this mutation makes the virus more infectious, it does not seem to alter the severity of the disease [26].

The mutation “ORF1b:P314L”, also known as “NSP12:P323L” [24], occurs in an open reading frame (ORF) region, ORF1b, which is responsible, together with ORF1a, for synthesizing the 16 non-structural proteins (NSPs) of the new coronavirus [27]. About NSP, the mutation occurs in NSP12, also called RNA-dependent RNA polymerases (RdRps), which are responsible for the synthesis of viral RNA [25]. Thus, the mutation occurs at position 323 of the 12th NSP, where proline (P) is replaced by leucine (L) [24]. Due to the high percentage of events of these two mutations and based on natural selection, it can be inferred that at least one of them contributes to the increased transmissibility of SARS-CoV-2.

**Figure 6:**
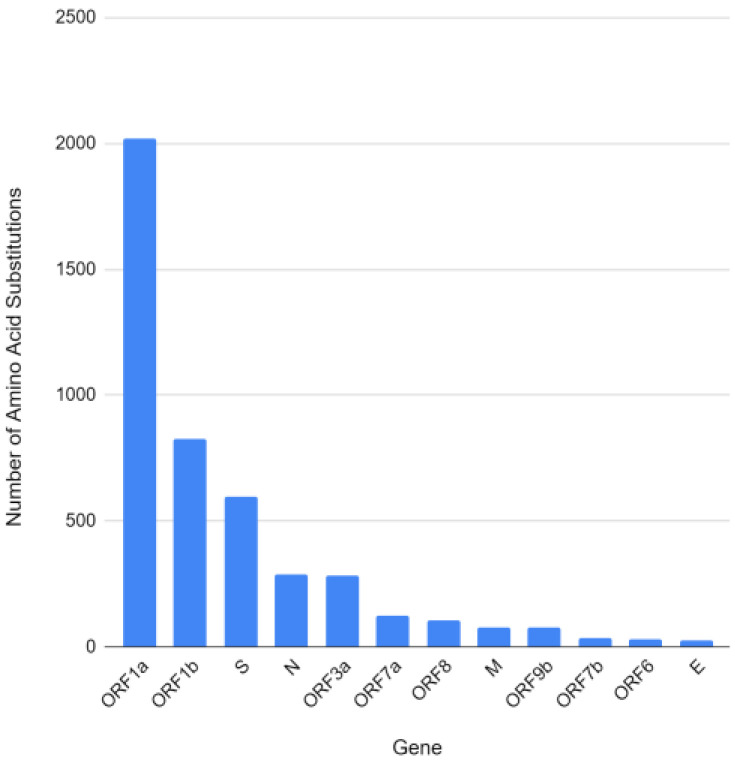
Number of amino acid substitutions in each of the coding regions of SARS-CoV-2.

The second analysis of genomic evaluation refers to the number of amino acid substitutions in each of the coding regions of SARS-CoV-2. The frequency of amino acid substitutions, concerning the frequency, randomly expected, decreases linearly with the increase in the physical–chemical differences between the pairs of amino acids involved in a substitution; in other words, the substitution frequencies may be dependent or independent of the physical–chemical parameters, the greater the difference between chemical properties, the greater probability of amino acid substitution being attributed to natural selection [28]. In this analysis, only the first time a given mutation occurred was considered; recurrences in other samples were not considered. These results are in Figure 6, in which the x-axis represents the mutation count and the y-axis represents the genes.

The ORF1a gene is, with a large difference from the others analyzed, the gene with the highest number of mutations, probably due to the size of its coding region. ORF1a is the largest gene of SARS-CoV-2, containing 13,202 nucleotides. In the analysis of the strains concerning quantity, the non-structural proteins that form the replication/transcription complex are encoded by genomic mRNA, where two overlapping ORFs are found. In the ORF1a region, a section encoding polyprotein 1a (pp1a) cleaved by viral proteases in each gene, the presence of 11 non-structural proteins (nsp1–nsp11) was detected. In the ORF1b region, the production of five additional non-structural proteins (nsp12–16) is observed [29].

The genome of the disease of the new coronavirus (COVID-19) was first sequenced in January 2020, approximately a month after its emergence in Wuhan, the capital of Hubei Province, China. In this regard, SARS-CoV-2 genome sequencing is essential to understand the behavior of the virus itself, its origin, the speed at which it mutates, and the development of drugs/vaccines and effective preventive strategies [30].

**Figure 7:**
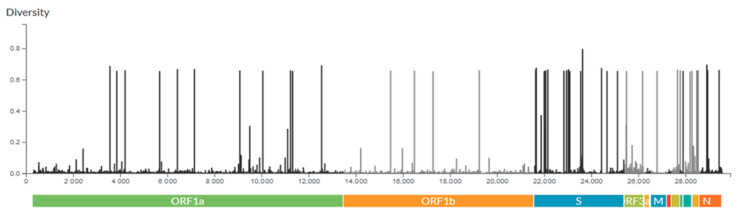
Entropy of genome information.

From genomic analyses, it was possible to observe the entropy of genome information of strains of SARS-CoV-2 evidenced in this study by Figure 7. The abscissa axis represents the 29903 nucleotides that make up the new coronavirus, in addition to the markings indicating the main coding regions of the virus. In the sequence, from left to right, are: ORF1a, ORF1b, S, ORF3a, E, M, ORF6, ORF7a, ORF7b, ORF8, ORF9b, and N. The y-axis of the graph exhibits the entropy of the information, which refers to how mutated a given region can be represented. Therefore, the larger the bar, the more amino acid substitutions occurred in that region.

**Figure 8:**
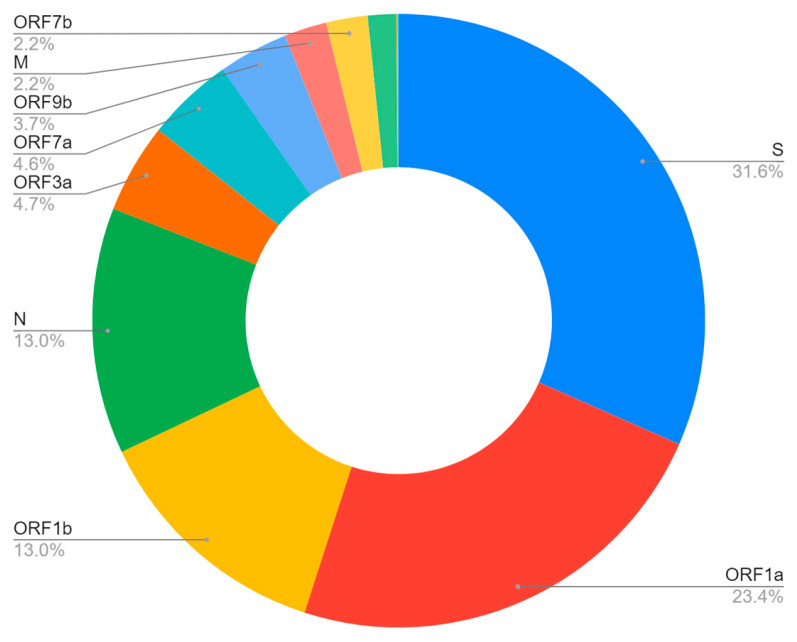
Percentage of mutation in each coding region.

As previously mentioned, in addition to ORF1a being the largest gene of SARS-CoV-2, a larger number of data is visible in the ORF1a region, followed by ORF1b and S. In this regard, the number of mutations is proportional to the number of bars in the graph; then, this perception corroborates with the previous result. According to the analysis of the entropy density in each of the genes, it is noticed that the S protein has the highest density; consequently, it is expected that this gene is the one that sustains most of the mutations [22]. The analysis in Figure 8 shows the percentage of mutation in each coding region, in which, unlike the previous analysis, amino acid substitution recurrences were counted.

During the pandemic period, the Brazilian healthcare system had several chronic problems with financing, management, provision of information, and structuring of services in general. However, because of the lack of financial management and advances in denialism, the variants were not fully controlled, causing greater mutations in Brazilian territory and diversified strains, even presenting one of the largest free universal health systems in the world [31]. Thus, it is necessary to evaluate the Brazilian strains in the phylogenetic tree system.

**Figure 9:**
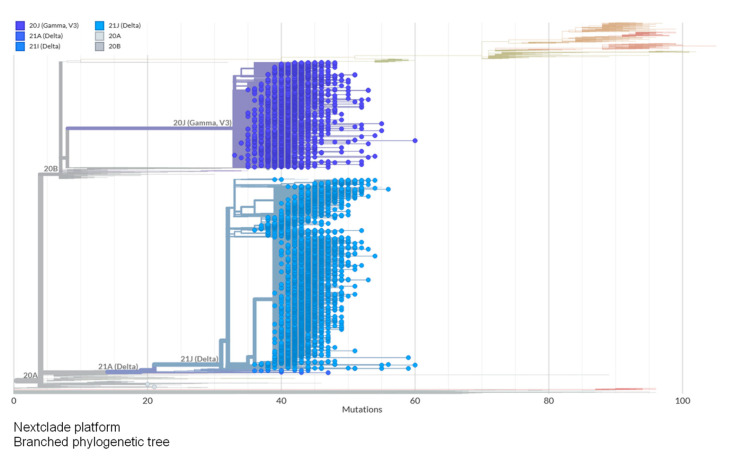
Phylogenetic tree of SARS-CoV-2 variants.

Figure 9 shows the phylogenetic tree of SARS-CoV-2 strains circulating in Brazil between June and November 2021. From this graphic, it can be pointed out that there is a very representative divergence between the strains of origin (Wuhan, China) either by the number of mutations or the diversity among the subvariants derived from other mutations in key sites.

The phylogenetic tree shows that between June and November (2021) only delta and gamma variants circulated in Brazil. It is also possible to observe that despite the phylogenetic difference between these two variants, they have approximately the same divergence. In this context, a relevant factor for the emergence of new viral variants is selective pressure caused by the abuse of antiviral drugs without proven efficacy [32,33].

It is a common phenomenon to be observed in developing countries the spread of alternative treatments regarding infectious diseases due to the precariousness of the health system and the fragility of the most economically vulnerable social strata [34]. The adoption of supposedly effective measures is often motivated by economic factors, naivety, or even scientific denialism. These reasons undermine the efforts of public health watchdogs not only at the local or regional level but on a national and international scale because the control of the movement of persons is practically unfeasible [35].

In this situation of neglect, with measures to prevent the spread of the virus, dissemination of information that generates doubt on the effectiveness of vaccination and testing methods, and use of drugs that do not have an antiviral activity, such as hydroxychloroquine (used for chronic inflammations and against malaria), ivermectin (antiparasitic drug), antibiotics of the class of macrolides, and vitamins in general, it is possible to trace a direct correlation with the emergence of new mutations and, later, new subvariants of SARS-CoV-2 [36].

Several scientific articles already published corroborate the data provided by this article; both regarding the symptomatic question and age group were evidenced both in Brazil and in other countries. According to Lima [37], in China, based on a study of 55,924 cases that were confirmed of COVID-19, fever and cough were the most common symptoms, accompanied by fatigue, sputum production, dyspnea, sore throat, headache, myalgia, chills, nausea, nasal congestion, diarrhea, hemoptysis, and conjunctival congestion, in this order. According to the same author [37], among the first 99 patients hospitalized and evaluated in a Wuhan hospital, the highest rate came from individuals over 50 years of age male.

Sousa Neto et al. [38] evidenced a study focused on three pillars (adults, symptomatic manifestations, and COVID-19) where they also brought fever and normal or dry cough as symptoms associated with the disease, which was demonstrated in the present trial using Figure 1, Figure 3 and Figure 4. Regarding the symptomatic gender group of COVID-19, according to a study conducted in the state of Rondônia, Brazil, deaths occurred equally until 40 years of age; however, above this age, the predominance of deaths came from the male sex [39]. Deaths for people over 60 years of age occurred at a prevalence of 52.7%, which is more than half of the cases [39].

Finally, the study of the genome of COVID-19 was relevant regarding the importance of the present study, considering the knowledge of the types of variants and their genetic sequences that can be the threshold between a rapid recovery or a large transmission and the need to study which and how many mutations the SARS-CoV-2 genes can perform in a given time interval [40,41]. Michelon [42] considers that mutations in the S protein are the most relevant considering clinical–epidemiological outcomes, due to their role in the infectious process, since it is a large transmembrane glycoprotein present on the external surface of coronavirus. According to the author, the affinity of binding to the cell receptor characterizes greater transmission, not different from that demonstrated in this article employing graphs of genomic mutations of SARS-CoV-2.

### 3.3. Immunological Factors

SARS-CoV-2 is responsible for the development of acute respiratory syndrome, aggravated by symptoms: cough, headache, and respiratory failures, which affect the entire oxygen absorption system. These symptoms are characteristic of the immune system process and essential to controlling and eliminating coronavirus infection [43].

The immune response to COVID-19 infection can be verified through rapid or serological tests. Serological tests for diagnosis of COVID-19 are based on the binding of antigens to antibodies, with different types of approaches, which can detect antibodies or viral antigens. The versions of commercial kits that detect antibodies against SARS-CoV-2 antigens that are mostly supplied on the market as IgG, IgM, or total antibodies (IgG + IgM) classes found in materials from the patient’s total blood, serum, or plasma samples [44].

However, the accuracy of serological tests varies by several factors, such as the implied methodology, antigen employed, and time of collection (ideally between the 3rd and 10th days for IgM, IgA, and total antibodies and, after the 15th day, for IgG). In addition, other factors may lead to errors in test results, such as immunosenescence (state of unregulated immune function in the elderly that contributes to increased susceptibility to infections) [45] and the immunological window (period where serological tests are not able to detect the immune response).

Thus, both class M (IgM) and class G (IgG) immunoglobulin antibodies can be detected seven days after the onset of clinical symptoms and may extend for more than 25 days, although the individual does not allow the individual to remain infectious, depending on his viral load and clinical presentation. The IgM peak is 5 to 12 days after SARS-CoV-2 infection, followed by a slow fall. IgG antibody reaches maximum concentrations after the 20th, approximately, as IgM antibodies disappear [46].

In addition, certain diagnostic changes may also be correlated with the vaccines that had been administered to patients. Depending on the technology used, vaccines are manufactured in different approaches, causing different antibodies produced by the immune system.

An approach to vaccine development is the creation of biological preparations derived from viruses cultured in vitro that are subsequently chemically inactivated, as evidenced in the CoronaVac vaccine (SinoVac Biotech, China) [47]. In addition, another known technology is viral vector vaccines, which use viruses deficient in replication, designed to express the genetic sequence of antigens of interest in host cells, such as Fiocruz (Oswaldo Cruz Foundation, Rio de Janeiro, Brazil). Additionally, vaccines manufactured by Janssen (Johnson & Johnson, Belgium) and AstraZeneca laboratories (University of Oxford, England) use the same approach but evidence the spike protein [48]. In addition, vaccines can also use mRNA, in which lipid nanoparticles are used to protect the mRNA encoding the prefusion-stabilized S protein on the way to intracellular space, as observed in the Pfizer vaccine (USA) [49].

Depending on the technical approach used, the coding protein may not be identified in the tests, in which protein S is generally the most prevalent in the tests available in retail chains of pharmacies and health care. It is more likely to be identified, providing a positive diagnosis [50]. However, some vaccines do not have protein S in their constitution, which are specific antibodies that would be identified in IgG and IgM tests; specifically, the viral spicule protein (S) are not present in the individual’s body. This fact does not necessarily mean that the individual is without immunological response, but only stopped external factors that provided non-stimulation for the production of specific immunoglobulin. In sum, this factor presented is also a possible impasse for the diagnosis of IgG and IgM serological tests [51,52,53].

A putative association between vaccination and the beginning of autoimmune disorders or immune-mediated phenomena, which have a complicated multifactorial origin, has also been examined and observed throughout time in relation to vaccines. It has recently been demonstrated that infections have a role in the etiology of autoimmune disorders, although vaccinations very rarely do. The patient may be protected by vaccinations not only from infectious illnesses but also from their consequences, such as autoimmune disorders [54].

Another determining agent for immunological factors is the SARS-CoV-2 mutations and variants, which may compromise information pre-established by the immune system [55]. After the emergence of the various waves of COVID-19 variants (respectively, alpha, beta, and delta), showing greater transmissibility and antibody escape capacity, substantial adaptability mechanisms were found against the actions of neutralizing antibodies induced by vaccination or previous immunization against a former variant [56,57]. In other words, antibody titers did not have such efficacy against new variants, as well as serological tests, which proved to be more suitable only for strains that did not undergo so many mutations in specific regions of detection of the tests.

### 3.4. Humanistic Perspective

A very pertinent point to be addressed is the rate of underreporting cases of COVID-19 and other flu-like syndromes in this pandemic period. It was observed in specific studies that the notification rate of COVID-19 in Brazil was estimated at 9.2% (95% CI: 8.8–9.5%), and in all states, the rates found were lower than 30%. São Paulo and Rio de Janeiro, the most populous states in the country, exhibited low notification rates (8.9% and 7.2%, respectively) [58]. The Brazilian population, within the period described, did not have full access to information and knowledge, and the notification rates were extremely low compared to other countries, a fact that follows the low population testing [59,60].

Given that citizens have not been warned, the large information deficit is affirmable and salient, resulting in denialism, which translates into the acceptance of interventions without scientific validation, such as the dissemination and exaltation of an unproven efficacy therapy with extremely serious side effects, such as chloroquine, or the defense of an intervention strategy that contradicts the position of the World Health Organization (WHO), named as “vertical isolation” [61,62].

Moreover, the Brazilian government in the period, actively promoted information by several vehicles (public and private) of a contrary position to the recommendation of the most respected world health surveillance agencies, and dissemination of the so-called fake news since the beginning [63]. Finally, the government’s failures statistically proved the increase in the number of deaths due to COVID-19, which could have been avoided in the light of a better administration [64].

In this scenario, the pandemic by COVID-19 proven to be a public health emergency of international interest and represented one of the greatest challenges to humanity and science since World War II. The interface of this problem with the aspects of mental health and psychological resilience necessary for health professionals is also of fundamental importance, during and after the pandemic crisis [65,66]. In the hospital area, they report such despair and suffering in the face of the neglect experienced; however, the government again demonstrated itself powerlessly [67].

From the One Health perspective, the pandemic has had impacts on the triad, both in the field of animal, environmental, and primarily human health, causing direct, and somewhat irreversible, damage to the country [68,69]. Furthermore, any effective global response to infectious diseases will require an appropriate institutional scenario, highlighting the importance of health knowledge areas being interconnected to assist governments and the population in the pandemic context [70,71].

## 4. Conclusions

The symptomatologic analysis of the patients demonstrated the highest incidence of cough (54%), headache (50%), and rhinorrhea (43%), with higher average reports of four, five, or six symptoms together (25.4%), emphasizing the symptomatic diversity that the SARS-CoV-2 can cause. Moreover, the profile of the participants was also delineated in most women, aged between 35 and 60 years, with a high unvaccinated rate.

The results of serological tests were correlated with the symptomatology of the participants highlighting the correspondence with seasonal infectious/inflammatory diseases, a fact that aggravated the precarious situation of hospitals and health centers. The genome of the virus was also evaluated within the months preceding the peak of infections by the omicron variant, underlining a higher incidence and rate of mutations in the spike protein encoders and ORFs. Furthermore, the strains were analyzed for phylogeny and ancestry to provide a better understanding of viral evolution.

In summary, these data provided essential reflections on the historical context and changes in the social and political scope. Remarkably, the symptomatologic, immunological, and genetic analyses redirect the Brazilian scenario in a global context, evidencing that the support of prophylactic measures ensures better management of health crises by infectious agents, both from the present and future perspectives.

## Figures and Tables

**Table 1:** Viral and bacterial agents causing symptoms similar to COVID-19.

Viral Agents			Bacterial Agents
Adenovirus	Enterovirus	Parainfluenza virus	*Chlamydophila pneumoniae*
Bocavirus	Influenza	Rhinovirus	*Mycoplasma pneumoniae*
Coronavirus (except SARS-CoV-2)	Metapneumovirus	Respiratory Syncytial virus (RSV)	*Mycobacterium tuberculosis*

Adapted from Borghetti et al. (2020) [20].

## Data Availability

Unrevised data was previously available at: https://www.medrxiv.org/content/10.1101/2022.11.12.22282248v1 (accessed on 24 January 2023).
